# The Upside of Negative Emotions: How Do Older Adults From Different Cultures Challenge Their Self-Growth During the COVID-19 Pandemic?

**DOI:** 10.3389/fpsyg.2022.648078

**Published:** 2022-05-09

**Authors:** Sofia von Humboldt, Neyda Ma. Mendoza-Ruvalcaba, Elva Dolores Arias-Merino, José Alberto Ribeiro-Gonçalves, Emilia Cabras, Gail Low, Isabel Leal

**Affiliations:** ^1^William James Center for Research, ISPA – Instituto Universitário, Lisbon, Portugal; ^2^Health Sciences Division, Universidad de Guadalajara CUTONALA, Guadalajara, Mexico; ^3^Department of Public Health, Universidad de Guadalajara CUCS, Guadalajara, Mexico; ^4^Departamento de Educación, Universidad Antonio de Nebrija, Madrid, Spain; ^5^Faculty of Nursing, University of Alberta, Edmonton, AB, Canada

**Keywords:** cross-cultural, negative emotions, older adults, self-growth, qualitative research, Covid-19 pandemic, positive psychology

## Abstract

**Background and Objective:**

The outbreak of Coronavirus Disease 2019 (COVID-19) has raised increased challenges for older adults’ personal growth in diverse cultural settings. The aim of this study was to analyze negative emotions and their role on older adults’ self-growth in Mexico, Italy, Portugal, and Spain, during the COVID-19 pandemic. For this purpose, a cross-national qualitative research was carried out.

**Methods:**

Data were collected from 338 community-dwelling participants aged 65 years and older, using a semi-structured interview protocol. Older adults were asked about negative emotions that significantly contribute to their self-growth during the COVID-19 pandemic. Content analysis was used to identify key themes.

**Results:**

Seven main negative emotions (fear, sadness, anger, grief, boredom, loneliness, and shame) significantly contributed to seven themes of self-growth, across the samples: sharing difficult experiences with others, supportive partner, spiritual practices, engagement with life, generativity, volunteering activities, and intimacy and sexual satisfaction. Sharing difficult experiences with others was most pertinent to Mexican (13.9%) and to Italian (3.0%) participants, and a supportive partner to Portuguese (12.1%), and to Spanish participants (6.5%).

**Conclusion:**

The findings of this study indicate that negative emotions during the COVID-19 pandemic contributed to their older adults’ self-growth. This study highlighted the cultural diversity of experiences during the pandemics and underlined the upside of negative emotions and its relation to older adults’ self-growth during this period.

## Introduction

The COVID-19 pandemic has had a significant impact on worldwide health, in particular among the older population. Globally, there were 76,382,044 confirmed cases of people infected by Coronavirus Disease 2019 (COVID-19), as of December 23rd, 2020 (World Health Organization, 2020a). By the end of 2020, the total number of cases registered by Portugal, Spain, Italy and Mexico was 5,533,106 (Italy = 1,977,370; Mexico = 1,338,426; Portugal = 378,656; Spain = 1,838,654) (Worldometer, 2020). The mortality rate of older adults with COVID-19 is higher than for individuals of other ages, as they are more likely to progress to a critical respiratory health status. Worldwide, the percentage of deaths increases with age, with 75% of decendents being 65 + years of age (World Health Organization, 2020b) and the number of deaths at these ages reaching 1,643,339 (World Health Organization, 2020a). In the United States, the country most affected by the pandemic, 14.8% of infected persons were aged 65 years and older (Centers for Disease Control and Prevention, 2020b). The COVID-19 pandemic has had a profound effect on the European older population: in the EU Member States, those aged 70 years and older accounted for 96% of the 168,000 additional recorded deaths between weeks 10 and 26 of 2020, when compared with the recent 4-year average (2016–2019) (Eurostat, 2020).

The COVID-19 pandemic has challenged older adults in relation to their negative emotions, by influencing their lifestyle, autonomy, the care and support they receive, their ability to remain socially connected, and how they are perceived by others (World Health Organization, 2020c). This population faces several challenges, such as spending more time at home; lack of physical contact with family, friends, and colleagues; temporary breaks from work and other activities; and anxiety and a fear of illness and death in relation to the self and to significant others (World Health Organization, 2020c).

The pandemic had a significant influence on the physical and psychological health of individuals and fostered negative emotions, such as fear, anger, grief, and bereavement, boredom, and shame. In addition, older adults expressed frustration, psychological problems, sleep problems, suicidal ideations, and loneliness (Mukhtar, 2020).

Indeed, the COVID-19 pandemic has been particularly harsh on the older population because of the frequent need for social isolation. Santini et al. (2020) found that the risk of depression and anxiety in older adults is higher when they do not have a social contact. Moreover, self-isolation affects older individuals who do not have social interaction at home. Those who have the support of voluntary services or social assistance, due to the absence of family or friends, may be at extra risk, as well as those who were already lonely or isolated (Armitage and Nellums, 2020). Moreover, given the increased likelihood of mental, autoimmune, cardiovascular, and neurocognitive problems in advanced life, social isolation among older individuals is a relevant topic for public health (Gerst-Emerson and Jayawardhana, 2015). As COVID-19 causes high mortality rates among the older population, the experience of death of significant others is an added challenge for isolated older adults (Armitage and Nellums, 2020).

The outbreak of COVID-19 has also raised major challenges for mental health services aimed at older adults in the community. Older adults have expressed their psychological frailty, as infection numbers continue to increase, as related both to their health and their importance to others (von Humboldt et al., 2022). In contrast, restrictions on public transportation to reduce the risk of disease transmission led to the widespread adoption of online mental health services (Liu et al., 2020). However, these services are only available to a small fraction of older people. Hence, the COVID-19 pandemic has become a major obstacle for older people’s access to treatment (Yang et al., 2020).

Casale and Flett (2020) discussed the importance of fear of being infected, especially relevant during the pandemic, and how it is apparently more pertinent now than other fears, such as the fear of loss or negative assessment. Due to the current global health crisis, fear has been felt by older people who become isolated or socially disconcerted, especially in relation to being forgotten by others in their usual social circles (Casale and Flett, 2020).

Moreover, chronological age is listed as a decisive factor in determining which of two people with an equal need should receive a ventilator in hospital settings (Fraser et al., 2020). Due to the policies that define all older people as equal (not reflecting heterogeneity), they end up feeling ashamed, and less significant as individuals (Fraser et al., 2020). Furthermore, as the virus continued to spread, the behavioral guidelines were constantly changed in light of rising morbidity and mortality rates, which may evoke hopelessness, confusion, anger, and fear (Fraser et al., 2020).

Psychological suffering, in particular anger, shame, sadness, and fear, is a function of the negative influence of this pandemic on the capacity of older adults to meet their most basic needs, such as participation in meaningful activities, physical and financial security, and social connection (Sanderson et al., 2020). However, it is important to note that although fear was predominately felt during COVID-19, sadness is also said to be an emotional state in need of treatment and over time (Sanderson et al., 2020).

Self-growth has been conceptualized as a dimension of psychological well-being that people of any age tend to accentuate as an important component in their lives (Bauer and Park, 2010; Ryff, 2014). Self-growth can be defined as experiencing continuous development and realizing its potential, while being open to new experiences and negative emotions that can potentially challenge points of view, and continuing to seek self-improvement (Ryff, 2014).

Although many people in advanced life continue to experience personal growth, others have a significant loss of capacity and require substantial care (World Health Organization, 2015). Continuous self-growth – mental, physical, social, and emotional – is important to enable older people to do what they value, and the ability to make decisions is the key to older people’s sense of control (Stephens et al., 2015). In addition, a broader view of the role of health and social care professionals includes their help in dealing with the negative issues of aging, such as physical decline and social loss, but also to promote self-growth and development even in the face of these associated negative aspects aging (Gladman, 2019).

Aging is naturally associated with undesirable issues of physical decline and social loss, but it is also associated with the positive aspects of self-growth and development, which are more likely when people have hope, are not alone, and maintain health (Steverink et al., 2001; Gladman, 2019). Self-growth and development are also associated with performing new activities, including during the pandemic. However, some older adults tend to avoid new challenges. A key role for health and social care teams is to help patients adopt new activities and to create opportunities to do so, even in the face of physical and mental disability (Nagayama et al., 2016). Moreover, self-growth is essential in the lives of older adults and is associated with a variety of wellness outcomes. Having positive relationships seems to become increasingly important for older adults to maintain greater self-growth (Toyama et al., 2020), for example through online technologies (von Humboldt et al., 2018; Kamin et al., 2020). Fasbender et al. (2014) indicated that older adults who perceive their own aging process as an opportunity for self-growth are more likely to work after retirement, which suggests that older adults seem to see work as a potential source of self-growth. In addition, Bauer and Park (2010) anticipated that older adults would focus more on intrinsically motivated human concerns, such as cultivating meaningful relationships and experiences. Thus, promoting positive relationships with other people can be a potential base or source for self-growth, especially in adulthood (Toyama et al., 2020).

Self-growth includes accepting negative emotions, health problems, and losses and developing a sense of accomplishment in life (Gladman, 2019). The perceptions of self-growth for older individuals have been associated with difficult experiences and emotions, and yet the COVID-19 pandemic offered these older people the opportunity to reflect and experience new perspectives of self-growth. For example, some participants reported self-growth by expanding their competences, for example by learning new technologies available online (von Humboldt et al., 2018, 2021). Moreover, literature indicates self-growth is related to age and culture (Hupkens et al., 2018).

To date, there is a lack of studies assessing negative emotions and their relation with self-growth among older adults and across cultures during the COVID-19 pandemic. However, in one study, old people reported an increase in negative emotions and levels of psychiatric morbidity during the SARS outbreak (Leung et al., 2003; van Bortel et al., 2016). Hence, research is needed in order to explore psychological and emotional responses among older adults. In this context, the objective of this study was to analyze negative emotions and their role on self-growth in older adults from Mexico, Italy, Portugal, and Spain, during the COVID-19 pandemic.

## Materials and Methods

### Recruitment and Sampling

This research has a cross-sectional multicenter cross-cultural study design. The sampling process started with strengthening previously existing community partnerships. The sample was recruited through direct contact with the older community-dwelling adults and senior universities and community centers, with which researchers had previous contact, in the study’s different countries. Institutions were located in Guadalajara, Cantanhede, Lisbon, Madrid, Milan, Turin, Genoa, and Cagliari. First, participants gave their informed consent to provide a telephone or online contact and answer an online questionnaire (e.g., Skype, Survey Monkey, Zoom, and Whatsapp). Following 395 initial contacts with the study’s target population, purposeful sampling resulted in a total of 338 Mexican, Portuguese, Spanish, and Italian community-dwelling older adults. Of the initial contacts, 33 decided not to participate due to lack of availability. Additionally, 24 were excluded, as they did not meet one or more of the following exclusion criteria: less than 65 years old; not clearly understanding the decision to participate in the study; and a history of psychiatric or neurological illness, or history of drug or alcohol abuse, which might compromise cognitive function (see Table 1). The four countries for this study were selected because they were affected by COVID-19 approximately at the same time.

**TABLE 1:** Sample socio-demographic and health characteristics.

Characteristics	Spanish 81 (24)	Mexican 94 (27.8)	Portuguese 98 (29)	Italian 65 (19.2)	Total 338 (100.0)
Age, mean ± SD	68.5 ± 4.03	69.7 ± 2.6	76.2 ± 4.1	69.7 ± 4.4	71.1 ± 3.4
**Gender, n (%)**					
Women	55 (67.9)	65 (69.1)	64 (65.3)	33 (50.8)	217 (64.2)
Men	26 (32.1)	29 (30.9)	34 (34.7)	32 (49.2)	121 (35.8)
**Education n (%)**					
Primary school	25 (30.9)	41 (43.6)	33 (33.7)	13 (20.0)	112 (33.1)
Middle school	22 (27.1)	22 (23.4)	24 (24.5)	35 (53.8)	103 (30.5)
≥ High school	34 (42.0)	31 (33.0)	41 (41.8)	17 (26.2)	123 (36.4)
**Marital Status n (%)**					
Married or in a relationship	58 (71.6)	61 (64.9)	54 (55.1)	48 (73.8)	221 (65.4)
Not married or in relationship	23 (28.4)	33 (35.1)	44 (44.9)	17 (26.2)	117 (34.6)
**Family Annual Income n (%)**					
≤ 25,000 €	29 (35.8)	72 (76.6)	76 (77.6)	17 (26.2)	194 (57.4)
> 25,000 €	52 (64.1)	22 (23.4)	22 (22.4)	48 (73.8)	144 (42.6)
**Perceived Health n (%)**					
Good	74 (91.3)	73 (77.7)	87 (88.8)	57 (87.7)	291 (82.3)
Poor	7 (8.7)	21 (22.3)	11 (11.2)	8 (12.3)	47 (17.7)

After the first online contact, using a semi-structured interview protocol, older adults were asked about negative emotions that significantly contributed to their self-growth during the COVID-19 pandemic. During this second moment of the data collection process, the interviewer took a mostly non-directive stance, and the topics covered in the interviews focused mainly on the challenges posed by their negative emotions and how the older population’s self-growth was expressed. Data sharing rules and participant consent were respected.

The interviews were conducted in an appropriate environment between May 1 and October 30, 2020. Sanitary recommendations to prevent COVID-19 infection were all respected in different countries. Mainly the physical distance during the interview and the use of a mask. The interview protocol was translated and made available in the study’s three languages (Italian, Portuguese, and Spanish). Interviews were implemented in the participant’s native language. The interview’s average duration was 35 min.

All participants responded to the same semi-structured interview protocol. All interviews were audio recorded and fully transcribed in a computer. After this data collection and transcription, interviews were submitted for in-depth reading and information analysis. All procedures were approved by the William James Center for Research Ethics Committee and ISPA – Instituto Universitário and were conducted in accordance with the ethical standards of the 1964 Helsinki Declaration and its subsequent amendments or comparable ethical standards.

### Data Analysis

Based on the information provided by the sample during the semi-structured interview protocol, an in-depth reading was undertaken to ensure that exhaustive contact with the data was made. Data were analyzed vis-à-vis content analysis (Erlingsson and Brysiewicz, 2017).

Initially, a code book was created for the purpose of content analysis. A code was assigned to each important category mentioned in the interviews. This system of codes later enabled a more complete and organized analysis. The coding procedure respected the rules of reliability and replicability, for example: codes applied consistently; mutually exclusive codes; and coding process implemented by more than one coder (Erlingsson and Brysiewicz, 2017). Three clinical psychologists independently codified all interviews. All such processes were found to be reliable (*k* = 0.87). The *p-value* considered for this study was ≤0.05, in all analyses.

After consensus was established in relation to main themes and subthemes, the codification process was exposed to a categorization procedure. Here, the main themes were grouped into clear and independent categories, discussed by consensus between the three coders. First of all, the thematic elements were isolated. Then, in order to organize and structure the collected data, these elements were reorganized and grouped. The system of categories thus developed was therefore an *a posteriori* categorization (bottom-up process), as categories were not established initially (Erlingsson and Brysiewicz, 2017). Category names were short and intuitive. To ensure a reliable and valid categorization system, general principles of classification and categorization of qualitative data were followed, such as: homogeneity, i.e., each set of categories was organized based on a principle common to its constituent elements, which remained fixed from the beginning to the end of the categorical classification; relevance, i.e., categories reflected the critical analysis of researchers, within the basic theoretical framework; and objectivity and fidelity, where the indexes defining the entry of elements into the categories were determined objectively, and these classification criteria were maintained until the end of the process.

**FIGURE 1:**
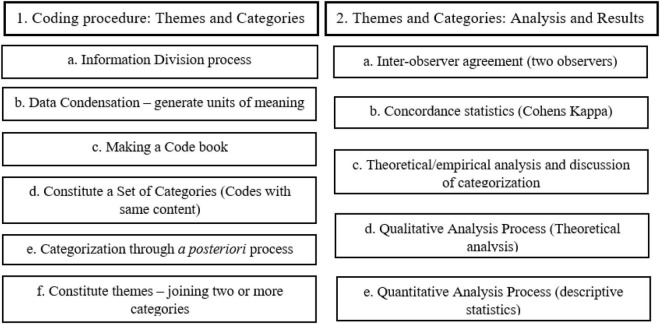
Adapted process of content analysis.

Finally, a matrix of interpretation of the results was developed, a fundamental step for the theoretical and empirical discussion of the obtained data. This process was divided into two parts: a quantitative descriptive analysis, with the calculation of means, percentages, frequencies, medians, and means of the sociodemographic variables, and a qualitative analysis of the information that emerged from the relationship between the theoretical models used and the empirical reality. The entire analysis process, for all interviews in all countries, is summarized in Figure 1.

## Results

The results indicated seven negative emotions: fear, sadness, anger, grief, boredom, loneliness, and shame. In addition, older participants described seven non-mutually exclusive themes that integrate different narratives about self-growth during the pandemic: sharing difficult experiences with others, supportive partner, spiritual practices, engagement with life, generativity, volunteering activities, and intimacy and sexual satisfaction. Although the word “challenged” was not always used explicitly, participant narratives indicated contexts and indicators consistent with the perception that their self-growth was challenged. The themes were comprehensive, unless otherwise indicated, and the information shared by each participant could contribute to different themes. All names are pseudonyms. All the participants (*n* = 338) verbalized negative emotions associated with self-growth in older age.

### Negative Emotion 1: Fear

A large number of participants (*n* = 50) indicated they felt fear during the pandemic. Portuguese participants reported this theme the most (*n* = 23 versus *nSpan* = 19; *nMex* = 8; *nItal* = 0).

During the pandemic, some participants considered negative emotions in a positive way, especially looking to future self-growth. Helena reported, “Sometimes I even think that fear, now with the pandemic, is a good thing. The fear of being infected forces us to stay at home and consequently we discover new things and try new things. I ended up learning a lot of things during the quarantine.” (Helena, female, 70 years of age).

### Negative Emotion 2: Sadness

Sadness was the next most prevalent negative emotion felt during the pandemic by older participants (*n* = 44). This theme was reported mainly by Spanish participants (*n* = 22 versus *nPort* = 16; *nItal* = 6; *nMex* = 0).

Although sadness is generally perceived as a negative emotion, some participants looked at sadness in a positive way. Duarte explained the following: “I often think that maybe this is how my life could end, due to a virus. I am really sad about it, but I have been through a lot and I have learned a lot, so I end up focusing on the good times of my life.” (Duarte, male, 84 years of age).

### Negative Emotion 3: Anger

The third negative feeling most felt by this sample of older adults was anger (*n* = 42), reported most often by Portuguese participants (*n* = 27 versus *nSpan* = 13; *nMex* = 1; *nItal* = 0).

In some cases, more intense feelings like anger developed during the quarantine. Filipe said, “At first it felt good to stay at home and rest, but after a while I started to get upset and angry, because I could never go out and get fresh air like before.” (Filipe, male, 82 years of age). Sergio added that “Most of the communication that arrives by cell phone is deceiving, fake news, which does not help.” (Sergio, male, 69 years of age).

### Negative Emotion 4: Grief

Some older adults (*n* = 41) mentioned grief as a negative emotion felt during COVID-19. Grief was reported mainly by Portuguese participants (*n* = 30 versus *nSpan* = 10; *nItal* = 1; *nMex* = 0).

The Coronavirus has been a major threat to older adults. However, when a family member or friend became infected, some older adults experienced grief. Beatrix explained: “I am an old woman, and I did not expect to feel grief again. But life is unpredictable and, sadly, I already had a friend who did not survive this terrible virus.” (Beatrix, woman, 85 years of age).

### Negative Emotion 5: Boredom

Boredom was the fifth theme most reported by the sample participants (*n* = 39), and mostly among by Mexican participants (*n* = 23 versus *nSpan* = 10; *nItal* = 6; *nPort* = 0). Boredom can be an emotion easily developed when isolation was imposed. As Antonio indicates “During the quarantine I did not go out, it is very difficult and boring to be at home” (Antonio, male, 65 years of age).

However, as Sara said, “I was bored always doing the same thing, until my grandson introduced me to the new social network: TikTok. I danced with her and I had a lot of fun” (Sara, female, 67 years of age).

### Negative Emotion 6: Loneliness

Some participants (*n* = 31) revealed they experienced loneliness during COVID-19, a theme reported most often by Mexican participants (*n* = 13 versus *nPort* = 12; *nSpan* = 8; *nItal* = 8).

Loneliness is a feeling commonly reported by the older population, and was further exacerbated during quarantine, when older people were forced into isolation. As reported by Luz “One feels isolated from what is happening in the world” (Luz, female, 74, years of age). Or as explained by Ana “I don’t spend much time with my children anymore and now with the pandemic I barely see them. We had some video calls, but it wasn’t the same. I wanted to hug them” (Ana, female, 79 years of age). Some participants, like Mariana, reported the upside of loneliness: “In this pandemic, it is also important to take advantage of loneliness, but since we are social beings we need communication” (Mariana, female, 70 years of age). Felipe added that “What I try to do is to go out and walk outside, because I get tired of being locked up. My children call me, but it is a just for a while. The rest of the time, I feel lonely” (Felipe, male, 88 years of age).

### Negative Emotion 7: Shame

The last negative emotion indicated in the study was shame (*n* = 12), indicated most often by Portuguese participants (*n* = 7 versus *nSpan* = 5; *nItal* = 0; *nMex* = 0).

Due to COVID-19, the older population had to be quarantined, bringing different emotions, including shame. David explained “Maybe it is unusual, but I felt ashamed of being at home with my wife and not knowing how to help her with the basic chores around the house. All my life I went to the office, did my work, and came home for dinner. Now, I stay at home and watch my wife doing many things and I don’t even know how to make lunch to thank her. But in the end I learned and I’m proud, because thanks to COVID-19 I learned to cook.” (David, male, 79 years of age).

All of the negative emotions felt by study participants were found to be significantly associated with seven major themes about self-growth: Sharing of difficult experiences with others, supportive partner, spiritual practices, engagement with life, generativity, volunteering activities, and intimacy and sexual satisfaction. The frequency attributed by each country to each theme and negative emotion was summarized in Table 2.

**TABLE 2:** Distribution by countries of negative emotions and main themes found.

Negative emotion	Spanish	Mexican	Portuguese	Italian
Fear	2	3	1	–
Sadness	1	–	2	3
Anger	2	3	1	–
Grief	2	–	1	3
Boredom	2	1	–	3
Loneliness	3	1	2	3
Shame	2	–	1	–
**Main themes**				
Sharing difficult experiences with others	2	1	2	3
Supportive partner	2	–	1	–
Spiritual practices	3	1	2	4
Engagement with life	2	1	4	3
Generativity	1	2	3	–
Volunteering activities	2	–	1	–
Intimacy and sexual satisfaction	2	–	1	–

*The numbers represent the order of importance/frequency that each country gave to each theme/emotion; 1 being the highest importance/frequency, 4 the lowest importance/frequency and “–” the absence of the theme/emotion.*

### Theme 1: Sharing Difficult Experiences With Others

Older participants (*n* = 81) indicated that sharing difficult experiences with other people is positively related to self-growth in later life, since others strive to overcome fear and sadness. This theme was mostly reported by Mexican participants (*n* = 47 versus *nPort* = 12; *nSpan* = 12; *nItal* = 10).

Older people often face the difficulty of a limited knowledge of technology. Diana stated that “Before this pandemic situation, my husband and I didn’t know anything about computers. We both had difficulties and we feared for our lives. But we both managed to learn a few things with the help of my grandson who worked at home.” (Diana, female, 76 years of age).

Some participants looked at the pandemic situation as a challenge. Jaime explained that “Throughout my life I have learned things from my wife. We both experienced new things and had moments together. We grew up together. And now the time has come for us to face sadness and overcome this phase of COVID-19 together” (Jaime, male, 82 years of age).

Participants reported that the situation was new for the entire population, which made it easier to share negative emotions with others. Laura verbalized that “we never went through a phase like this, where we all wear masks, clean our hands and spend most of our time at home. It is something new and we can all learn from each other. And together we can end this and get back to normal” (Laura, female, 71 years of age).

### Theme 2: Supportive Partner

A large part of the participants (*n* = 63) indicated that the presence of a supportive partner was advantageous for different negative emotions such as fear and shame, thus contributing to their self-growth. This theme was mostly reported by Portuguese participants (*n* = 41 versus *nSpan* = 22; *nMex* = 0; *nItal* = 0).

A supportive partner can help older adults in the most difficult moments, because they provide support to overcome them, contributing to their self-growth. Eric pointed out that, “I often stop believing this pandemic situation will end. But my wife is more optimistic than me and she helps me hope that one day soon we will go out again as before” (Eric, Male, 88 years of age).

There are also older people who believe that they can positively overcome the pandemic. Nadia said, “When I was younger, I was an insecure girl. But I have been married to my husband for many years and he helped me have more confidence in myself. I grew up and became a better person and now I can confidently say that we will be able to overcome this pandemic. With him, I don’t feel fear of the future” (Nadia, female, 77 years of age).

Both partners provide support, but at different times in life. Filipa said, “Finding a person who supports us and teaches us to be better people is rare. But in my case, I found one, and it’s my husband.” And she continued, “I am grateful for the person I have become and today I am the one who helps him deal with this sad situation (COVID-19)” (Filipa, female, 65 years of age).

### Theme 3: Spiritual Practices

Thirty-six participants verbalized that spiritual practices, for example, praying, meditating, and reading books about spirituality, were positively associated with self-growth in old age. Spirituality addressed anger and grief, in particular. This theme was reported mainly by Mexican participants (*n* = 12 versus *nPort* = 11; *nSpan* = 9; *nItal* = 4).

With the COVID-19 pandemic, older adults reported fear of being lonely and spiritual practices were relevant for challenging anger, grief, and feelings connected to the community. As reported by Hugo “Spirituality has played an increasingly important role in my life. And now more than ever, it is helping me to face grief because I am more focused on being with others,” reported Hugo (Hugo, male, 68 years of age). Elena added that “Thanks to the ministers and the brothers of the church, because they continued communicating activities and praying at a distance, I have felt calm, although it is not the same” (Elena, female, 68 years of age).

Spiritual practices changed the perspective of older adults regarding their current negative emotions. Anabela verbalized, “As soon as we connect with the spirit world, we see things differently and I see that we will be victorious in this fight against COVID-19. I don’t feel any fear or anger” (Anabela, female, 77 years of age).

For some participants, spirituality was an individualized experience. Maria explained that “No two people are alike and that is why no two experiences are alike. For me, spirituality helped me have hope and stop anger, gave meaning to life and consequently helped me grow. And I have faith. I have faith that this will end quickly” (Maria, female, 92 years of age).

### Theme 4: Engagement With Life

There were also some participants (*n* = 29) who reported that engagement with life was an activity that contributed to self-growth, due to the diversity of things they can do. This topic was most often mentioned by Mexican participants (*n* = 12 versus *nSpan* = 8; *nItal* = 7; *nPort* = 2). Engagement with life was used to tackle boredom during the pandemic. As reported by Juana “I like to watch TV, sometimes I look up my videos and movies, also on my cell phone, but I like it better on TV, and I like to watch movies, I spend time there, I don’t get bored” (Juana, female, 79 years of age).

Engagement with life was perceived in different ways. For some, this activity can mean new perspectives as Hugo mentions, “Usually when I talk about engagement with life, I quickly like to mention my adventures in other countries. I love to travel because I learn so much! But in this pandemic situation I had to leave that dream behind and live my adventures at home.” (Hugo, male, 67 years of age).

For other participants, this activity was more focused on quieter moments, such as enjoying time reading a book, doing the crossword in the magazines or even crochet. As Ritamaria verbalized, “I like to think that I live my adventures in my head. I read and imagine what I want. I definitely benefit from my self-growth through the diversity of stories I imagine.” (Ritamaria, female, 88 years of age).

On the other hand, older participants value their health and well-being, and in this way, many associate engagement with life activity with sport. As David said, “During the quarantine I had more time to play sports, something I really like. It makes me feel alive and young again. I learned and grew up doing sport and I won’t stop now.”(David, male, 74 years of age).

### Theme 5: Generativity

Generativity was reported by some participants (*n* = 23) as an important factor for older adult self-growth. This theme indicates a concern with guiding the next generation and was reported mainly by Spanish participants (*n* = 10 versus *nMex* = 7; *nPort* = 6; *nItal* = 0).

From the perspective of some older participants, the pandemic offered an opportunity to combat boredom, overcome shame and carry out long overdue activities. As Raul explained, “How many times do we tell ourselves “when I have time, I will do this”? Many others and I took advantage of all the time that COVID-19 gave us to develop our self-growth. In fact, I started writing a book that I wanted to have started a few years ago. I was bored and there you go” (Raul, male, 70 years of age). Rosa added that “I continued teaching classes, I was able to receive them, update myself, have contact with students, friends, family and patients, to participate in academic and friend meetings” (Rosa, female, 66 years of age).

Before we leave this world, we like to leave our mark and be remembered. As verbalized by Laura, “I cannot just leave this planet without making a contribution. I want my children to not feel ashamed of me. The pandemic is shortening my time and so I decided to teach my granddaughter math during the quarantine” (Laura, female, 65 years of age).

Some participants indicated that they left their contribution and felt very integrated in the community. Susan reported, “I was a university professor for many years. In fact, I taught, but at the same time I also learned from my students. Today I see publications by my former students about new jobs or new positions and I am proud that I had contributed in some way to their success.” (Susan, female, 67 years of age).

### Theme 6: Volunteering Activities

Twenty participants in this study indicated that volunteering activities are positively linked with being bored during the pandemic. This theme was reported mainly by Portuguese participants (*n* = 12 versus *nSpan* = 8; *nMex* = 0; *nItal* = 0).

Voluntary activities by older people brought them satisfaction, subsequently contributing to self-growth. As Paula reported, “I feel important when I help others with volunteer work. I am never bored. In addition, I also learn many things as a volunteer” (Paula, female, 68 years of age). Jorge added that “All this is helping me know and value my life more and the life of others.” (Jorge, male, 66 years of age).

Some older adults fought boredom by volunteering. As Raul said “I am aware there are advantages to doing volunteer work, because I did it for most of my life. But now I have to find other ways to grow and stop being bored in front of the TV” (Raul, male, 89 years of age).

Due to the pandemic, older people used technology for volunteering activities. Sam verbalized that “volunteering has become virtual for me. However, I don’t feel confident in this area, and I had to learn it during summer” (Sam, male, 65 years of age).

### Theme 7: Intimacy and Sexual Satisfaction

This theme was reported by 14 participants. Intimacy and sexual satisfaction – defined as the emotional and intimate experience of frequent and mutual sexual pleasure – was also considered an important factor to self-grown in old age. This theme was mostly reported by Portuguese participants (*n* = 10 versus *nSpan* = 4; *nMex* = 0; *nItal* = 0).

Many older individuals believe the sexual component is important for their life. A positive intimacy and sexual satisfaction were related to boredom and shame, which implied self-growth. Tatiana asked herself “How can we have a good sexual relationship if we don’t follow what makes us be alive? The truth is when we don’t have it, we have to fight shame and doing nothing, and to invest in ourselves with or partner.” (Tatiana, female, 69 years of age).

Besides that, older people are aware that sex is not acquired knowledge, but knowledge that is always in development. Brian explained, “None of us was born knowing how to do anything, least of all how to have sex. We learn. And this is how we develop self-growth.” (Brian, male, 82 years of age).

Intimacy and sexuality were considered important elements for older participants’ growth during the pandemic, as Rita reported, “A person’s self-growth implies being happy in the bedroom. Where else can you find a better way to spend your time when isolated at home? In bed with the one you love.” (Rita, female, 86 years of age).

## Discussion

The objective of this study was to analyze negative emotions and their role on older adults’ self-growth from a cross-cultural perspective. The findings indicate seven negative emotions (fear, sadness, anger, grief, boredom, loneliness and shame) were perceived as related to seven non-mutually exclusive themes of self-growth: sharing difficult experiences with others, supportive partner, spiritual practices, engagement with life, generativity, volunteering activities, and intimacy and sexual satisfaction.

Worldwide, there is an uncertainty and unpredictability associated with COVID-19, with this influencing people’s physical and mental health, particularly in terms of emotions. In addition, public health emergencies trigger more negative emotions and also affect cognitive abilities in older populations (Li et al., 2020). The COVID-19 pandemic has contributed to mental health concerns worldwide. In relation to this, although older adults may feel negative emotions during the pandemic, these can have an upside and therefore positive impacts (Li et al., 2020).

Many participants, most of whom were Portuguese participants, reported fear. Fear was positively related with having a supportive partner and sharing difficult experiences with others. These participants channeled fear into self-protection and safety behaviors, such as hygiene, social distancing, and avoiding going out as much as possible. Another example is the fight against feeling asphyxiated because of the mask, where fear prevents its removal (Sanderson et al., 2020). Participants with fear also showed hope that a vaccine would be possible. However, several general studies have shown that the vaccine’s effectiveness decreases significantly with age, which is related to the age-related progressive decline in innate and adaptive immune responses (Poland et al., 2018). During the first few months of the pandemic, these arguments about vaccines remained uncertain, but later the evidence has been clear that vaccine effectiveness for COVID-19 is effective in all age groups (Centers for Disease Control and Prevention, 2021a). Furthermore, themes such as volunteering activities were more frequent among Spanish and Portuguese older people. One of the reasons behind this difference is the European Union’s particular investments in the active aging policy in recent decades, which include various socialization and volunteering activities, stressing the importance of social contact among older adults and contributing to their good-being (European Comission, 2019). Intimacy and sexual satisfaction was also an important theme for the self-growth of Portuguese and Spanish older people. Although this theme is still a taboo among the older population in general, in Latin America, in some countries particularly, such as Mexico, strong negative beliefs and taboos about the sexuality of older adults, may decrease the importance attributed to this theme (Villanueva et al., 2020).

Sadness was reported as the second most prominent negative feeling and was mostly expressed by Spanish participants. This emotion was significantly related with having a supportive partner and sharing difficult experiences with others. Sadness may motivate an individual to engage in useful behavior in order to help restore or replace the loss, and is therefore also related to the feeling of grief. Grief was also significantly related to spiritual practices for these participants. As for the pandemic, transitory losses may be more frequent; however, the sadness that comes with losses may be a means to recovery. An example would be missing significant connections (e.g., children, grandchildren, pets, friends, or others) during social isolation (Sanderson et al., 2020).

Older participants, particularly Portuguese participants, experienced anger as well. Anger was significantly related to spiritual practices. Older adults were willing to escape painful feelings of shame, and thereby expressed guilt and anger as a convenient scapegoat. While blaming others can help individuals regain some sense of control and superiority in their lives, in the long run the costs tend to be perceived as non-satisfactory (Tangney et al., 2007).

The COVID-19 pandemic provided the opportunity to improve mental health, personal growth, and mindfulness through personal activities, fostering resilience and protection against a strong psychological impact (Mukhtar, 2020). Boredom was the fifth most expressed negative emotion and was mostly expressed by Mexican participants. Boredom was significantly related to intimacy and sexual satisfaction, engagement with life, generativity, and volunteering activities. Boredom generally stemmed from the perception of uncontrollable events and was circumvented through controlled actions, such as improving hygiene, healthy eating, sleep, physical exercise, meditation, painting, movement dance, cooking, language acquisition, knitting, gardening, reading books, listening to music, and watching films or series during the period of social isolation (Mukhtar, 2020). Moreover, the literature indicates that older adults may explore resilient and protective strategies to deal with boredom (Wood and Rünger, 2016).

Some participants, most of whom were Mexican participants, reported loneliness. Loneliness was significantly related to having a supportive partner and sharing difficult experiences with others, which corroborates the existing literature (Sanderson et al., 2020). The isolation of high-risk groups, such as older adults, has been highly recommended or mandatory, in order to reduce spread to these groups (Armitage and Nellums, 2020). But strategies chosen by older adult caregivers, to reduce the transmission and mortality caused by COVID-19, have contributed to the loneliness of this population (Patel and Clark-Ginsberg, 2020). Loneliness is associated with other negative feelings, such as depression, unhappiness, dissatisfaction with life, and thus can manifest itself in physical health (Golden et al., 2009; Patel and Clark-Ginsberg, 2020). To combat these feelings, there are social service programs for older adults that focus on personal social interactions. However, with COVID-19 and compulsory social distance, loneliness is expected to increase among the older population in the pandemic phase (Patel and Clark-Ginsberg, 2020).

Finally, shame was significantly related to generativity, and intimacy and sexual satisfaction. Although shame can promote isolation and withdrawal in older adults, causing separation from social resources, it can also encourage reflection, cognitive elaboration, and analysis of more difficult events (Walmsley and McCormack, 2016; Apesoa-Varano, 2018). Shame can also be a factor of empowerment and claim in older adults, allowing for self-growth (Apesoa-Varano, 2018).

Overall, we found that loneliness was the only negative emotion that was evidenced in participants from the four countries. This may most likely be due to isolation and the general distancing from the support and social networks that exist among older adults, who were labeled as the main population at risk of infection for COVID-19 (Centers for Disease Control and Prevention, 2021b). Moreover, before the pandemic, the CDC had already warned to the fact that loneliness was being one of the main widespread scourges of this century among the older population (Centers for Disease Control and Prevention, 2020a). Furthermore, it was found that Spanish and Portuguese participants consistently presented higher levels of fear, sadness, anger, grief and shame emotions, while Italians and Mexicans consistently showed lower levels of these emotions. This difference may be due to the strong and recognized value given to family coexistence in the Iberian Peninsula, which was castrated during the pandemic, and may have contributed to the growth of the intensity of these negative emotions (Cunha and Franco, 2010). The Spanish and Portuguese participants may have shown more negative emotions also due to the management and evolution of the pandemic. Indeed, older adults in Mexico and Italy had particularly drastic peaks of infection and social isolation, but later ended up regularizing, while the confinement measures were longer in Portugal and Spain; which may have caused greater emotional instability among older adults (Jornal de Negócios, 2020; BBC News Brazil, 2021).

We found relevant cross-cultural differences in the narrative of study participants, concerning themes that contribute to self-growth. Sharing difficult experiences with others was most pertinent to Mexican and Italian participants, while having a supportive partner mattered most to Portuguese and Spanish participants. As already mentioned, in the Iberian Peninsula, traditional family values are very strong, which may justify the importance of Spanish and Portuguese older adults for their partner’s support for self-growth (Cunha and Franco, 2010).

Sharing difficult experiences with others was the theme most mentioned by our studied sample, particularly among older Mexican persons. Sharing helped participants deal with fear and sadness. In the rapidly aging population, older adults are adapting to new technologies and the demands of modern society, in order to overcome the difficulties and limits of their social and emotional isolation. During the pandemic, the use of new technologies by the older population has a beneficial effect on their quality of life and meaning of life (Roupa et al., 2010; von Humboldt et al., 2018, 2021).

Technological innovations can play a significant role in promoting healthy aging and social participation in older age. However, how social contexts can influence or support the use of technology by older individuals is not well understood (Kamin et al., 2020). The study by Kamin et al. (2020) showed that, during the pandemic, supportive behaviors were associated with the current use of technology. Older participants used technology for sharing difficult experiences with others. Intelligent technology has been shown to play an important role in exploring self-growth, meaning in life, personal meaning, sense of agency, and self-management, as well as other person-centered developments in old age (Martel et al., 2018).

The second-most indicated theme by participants was having a supportive partner, mentioned most by Portuguese participants. The feelings of fear and shame were an investment in a supportive partner during the pandemic. Self-growth is generally considered to be a result of intrapersonal processes – personal resources that reside within the person (Lee et al., 2018). Lee et al. (2018) suggested that the link between supportive relationships and personal growth is relevant. Another study indicated that highly emotional experiences, such as romantic relationships, despite the range of emotions they produce and the problems they can cause, can often lead to important areas of self-growth in old age (Turner, 2016).

The next most reported theme was spiritual practices, most mentioned by Mexican participants. For these participants, spirituality addressed anger and grief. Spirituality has been associated with personal growth in the literature (Ryff, 2014). Although spirituality has been considered a construct similar to religiosity, the former has been considered an independent and more consistent predictor of self-growth and related psychosocial constructions (Ivtzan et al., 2013; Toyama et al., 2020). Older individuals acquire spiritual awareness and recognition, which provides a cultural power in their lives (Stanhope and Lancaster, 2013). In fact, as spiritual aspects become more intense (Ravanipour et al., 2013) greater perception of personal growth is reported (Hajinejad et al., 2019). The findings of Lifshitz et al. (2019) indicated that not all domains of spirituality are equally dominant in people’s lives or are positively associated with subjective well-being, and that encouraging older adults to develop their personal spirituality and personal growth can contribute to their well-being.

The fourth-most indicated theme by the participants was engagement with life, mentioned mostly by Mexican participants. Engagement with life was significantly related to boredom. The COVID-19 pandemic promoted negative emotions, and in this context older adults expressed frustration, which can be tackled with engaging activities (Mukhtar, 2020). Literature indicated that engagement with life is related to being receptive to different perspectives and activities, often related to older adults’ personal interests (Reichstadt et al., 2010; von Humboldt et al., 2013a,b, 2014; von Humboldt and Leal, 2014a,b, 2015b).

The fifth most indicated theme was generativity, mentioned mostly by the Spanish participants. This theme was reported as relevant for overcoming boredom and shame. Generativity can interact with gratitude to strengthen well-being. Given the positive relationship between generativity and age, more generative individuals experience higher levels of well-being and self-growth (Arnold and Clark, 2016; Portocarrero et al., 2020). Psychosocial factors, such as generativity, volunteering, positive interpersonal relationships, and spirituality, may affect the personal growth of older adults longitudinally (Toyama et al., 2020). Cultural differences in generativity may also be influenced by the economic context of older participants. Older ethnic minority groups have disproportionately less work-related income, are less likely to have retirement pensions, have fewer opportunities for being generative, have a higher incidence of chronic illness disease, and have higher health expenses (von Humboldt et al., 2013c,d; von Humboldt and Leal, 2015a; von Humboldt et al., 2021). As a result, older ethnic minority groups have, on average, greater health needs and fewer savings and resources (Ferreira-Valente et al., 2019; Davis and Willink, 2020; Portocarrero et al., 2020).

Volunteer activities was the next most reported theme which seemingly resonated most with Portuguese participants and addressed boredom. Volunteering in old age has been associated with personal growth in the literature (Ryff, 2014). Intimacy and sexual satisfaction was the least prevalent theme and was mostly mentioned by Portuguese participants. For these participants, intimacy and sexual satisfaction tackled boredom and shame. The literature points out the relevance of sexual well-being for strengthening relations and facing adversity in difficult moments (von Humboldt et al., 2021; Ribeiro-Gonçalves et al., 2022).

### Limitations and Strengths

The present study has a number of limitations. Results may have been subject to researchers’ bias, namely some influence of personal values on data analysis, although this process may not have been fully conscious for the researchers; hence the importance of a consensus among researchers regarding the themes and categories created during the analyzes (Creswell and Miller, 2000). Also, researchers with poor knowledge about the construct used in qualitative research can influence the richness of the analyzed contents (Creswell and Miller, 2000). In addition, this study showed a small sample size, questioning the representativeness of the data analyzed, although qualitative studies may have a more in-depth and comprehensive nature than other studies (Bridges et al., 2010). This study addressed the older population, whereby data collection was not succinct, but deep and detailed. Therefore major limitations could have arisen from sample fatigue and withdrawal from participation (Creswell and Miller, 2000; Poggenpoel and Myburgh, 2005; Stanziano et al., 2010; Phoenix, 2018).

Notwithstanding these limitations, this exploratory study is relevant for a number of reasons. Qualitative cross-cultural studies are scarce, particularly among older populations. This kind of study permitted us to identify positive and negative health-related dimensions in diverse contexts and in-depth. Qualitative cross-cultural studies can help researchers to identify variables that may influence self-growth, allowing the characterization of self-growth and negative symptoms in pandemic context to manifest in a more complete way (López et al., 2020; von Humboldt et al., 2021). In line with the literature, this study is relevant to the implementation of policies for psychosocial interventions that will be increasingly important as the pandemic impacts on the mental health of older adults (López et al., 2020; Monahan et al., 2020). The recruitment of the older population in different countries during a pandemic is made even more difficult as such persons have been deemed most at risk for COVID-19. Nonetheless, in addition to allowing clarification of conceptual inconsistencies arising from monocultural studies, researchers can identify the skills that older people stand to gain while adjusting to a pandemic in different contexts (Chen and Bonanno, 2020; López et al., 2020).

Assessing negative emotional states in order to better understand the self-growth process is important in a pandemic context (Reichstadt et al., 2010; Sun et al., 2021). Older patients receiving supportive health care treatments during COVID-19 report negative emotional symptoms (Sun et al., 2021) and also do so during the prophylactic process of social isolation (Smith et al., 2020). When people are infected with COVID-19 or are being prophylactically treated, they tend to feel unsupported, sad, lonely, wronged, and/or irritated. Along with the sense of imminent danger, adverse feelings can contribute to the process of elaborating contingency plans, promoting resiliency, and stimulating agency (Morrow-Howell et al., 2020; Smith et al., 2020; Tyrrell and Williams, 2020).

Negative emotions can promote negative states, but they can also be important sources of emotionally demanding contingency management resources, as seen in the context of the current pandemic (López et al., 2020; Morrow-Howell et al., 2020). In this context, this study highlights that negative emotional aspects associated with the pandemic can later be associated with positive aspects in the daily lives of older adults, promoting skills in several areas, such as stronger connections to family and friends, greater attention to self-care, greater comfort working with online platforms, greater care and respect for daily time management, and more willingness to fight isolation (Morrow-Howell et al., 2020). We consider that the results of this study can be linked to the positive view on aging, as explored by Tornstam (1997). Indeed, in his gerotranscendence theory, Tornstam (1997) highlighted that older adults tend to move away from a materialistic perspective and become more aware of their dimensions of self, cosmic energy and relationships shift. In this context, older adults choose to have time to themselves, to invest in their self-care and their well-being, and to be selective about meaningful relationships.

In brief, this preliminary study is relevant to the still unknown relation between negative emotions and self-growth among older adults from different cultures, stressing the relevance of sharing difficult experiences with others, a supportive partner, spiritual practices, engagement with life, generativity, volunteering activities, and intimacy and sexual satisfaction to tackle negative emotions, in old age.

## Data Availability Statement

The raw data supporting the conclusions of this article will be made available by the authors, without undue reservation.

## Ethics Statement

The studies involving human participants were reviewed and approved by William James Center for Research Ethics Committee and ISPA – Instituto Universitário. The patients/participants provided their written informed consent to participate in this study.

## Author Contributions

SH participated in the research design, data collection, data analysis, and manuscript elaboration and revision. NM-R and EC participated in the data collection, data analysis, and manuscript revision. EA-M participated in the data analysis and manuscript revision. JR-G participated in the manuscript elaboration. GL and IL participated in the manuscript revision. All authors contributed to the article and approved the submitted version.

## Conflict of Interest

The authors declare that the research was conducted in the absence of any commercial or financial relationships that could be construed as a potential conflict of interest.

## Publisher’s Note

All claims expressed in this article are solely those of the authors and do not necessarily represent those of their affiliated organizations, or those of the publisher, the editors and the reviewers. Any product that may be evaluated in this article, or claim that may be made by its manufacturer, is not guaranteed or endorsed by the publisher.
